# Tolerability and efficacy of vortioxetine versus SSRIs in elderly with major depression. Study protocol of the VESPA study: a pragmatic, multicentre, open-label, parallel-group, superiority, randomized trial

**DOI:** 10.1186/s13063-020-04460-6

**Published:** 2020-08-03

**Authors:** Giovanni Ostuzzi, Chiara Gastaldon, Angelo Barbato, Barbara D’Avanzo, Mauro Tettamanti, Igor Monti, Andrea Aguglia, Eugenio Aguglia, Maria Chiara Alessi, Mario Amore, Francesco Bartoli, Massimo Biondi, Paola Bortolaso, Camilla Callegari, Giuseppe Carrà, Rosangela Caruso, Simone Cavallotti, Cristina Crocamo, Armando D’Agostino, Pasquale De Fazio, Chiara Di Natale, Laura Giusti, Luigi Grassi, Giovanni Martinotti, Michela Nosé, Davide Papola, Marianna Purgato, Alessandro Rodolico, Rita Roncone, Lorenzo Tarsitani, Giulia Turrini, Elisa Zanini, Francesco Amaddeo, Mirella Ruggeri, Corrado Barbui

**Affiliations:** 1 Department of Neuroscience, Biomedicine and Movement Sciences, Section of Psychiatry, University of Verona, Verona, Italy; 2 Istituto di Ricerche Farmacologiche Mario Negri IRCCS, Milan, Italy; 3 Department of Neuroscience, Rehabilitation, Ophthalmology, Genetics Maternal and Child Health, University of Genoa, Genoa, Italy; 4 Section of Psychiatry, IRCCS Ospedale Policlinico San Martino, Genoa, Italy; 5 Department of Clinical and Experimental Medicine, Psychiatry Unit, University of Catania, U.O.C. Clinica Psichiatrica, A.O.U. Policlinico-Vittorio Emanuele, Presidio “G. Rodolico”, Catania, Italy; 6 Dipartimento di Neuroscienze, Imaging e Scienze Cliniche Università “G. D’Annunzio Chieti-Pescara”, Chieti, Italy; 7 Dipartimento di Medicina e Chirurgia, Università di Milano Bicocca, Milan, Italy; 8 Department of Human Neurosciences, Sapienza University of Rome, Rome, Italy; 9 Department of Medicine and Surgery, Division of Psychiatry, University of Insubria, Varese, Italy; 10 Division of Psychiatry, University College London, London, UK; 11 Institute of Psychiatry, Department of Biomedical and Specialty Surgical Sciences, University of Ferrara, Ferrara, Italy; 12 Department of Mental Health, ASST Santi Paolo e Carlo, Milan, Italy; 13 Department of Health Sciences, Università degli Studi di Milano, Milan, Italy; 14 Azienda Ospedaliera Universitaria Mater Domini, Università Magna Grecia, Catanzaro, Italy; 15 Dipartimento di Medicina Clinica, Sanità Pubblica, Scienze della Vita e dell’Ambiente, Università degli Studi dell’Aquila, L’Aquila, Italy

**Keywords:** Randomized clinical trial, Pragmatic trial, Elderly, Antidepressants, Vortioxetine, Depression

## Abstract

**Introduction:**

Depression is a highly prevalent condition in the elderly, with a vast impact on quality of life, life expectancy, and medical outcomes. Selective serotonin reuptake inhibitors (SSRIs) are the most commonly prescribed agents in this condition and, although generally safe, tolerability issues cannot be overlooked. Vortioxetine is an antidepressant with a novel mechanism of action. Based on studies to date, it may have a promising tolerability profile in the elderly, as it does not adversely affect psychomotor or cognitive performance and does not alter cardiovascular and endocrine parameters. The present study aims to assess the tolerability profile of vortioxetine in comparison with the SSRIs considered as a single group in elderly participants with depression. The rate of participants withdrawing from treatment due to adverse events after 6 months of follow up will be the primary outcome.

**Methods and analysis:**

This is a pragmatic, multicentre, open-label, parallel-group, superiority, randomized trial funded by the Italian Medicines Agency (AIFA - *Agenzia Italiana del Farmaco*). Thirteen Italian Community Psychiatric Services will consecutively enrol elderly participants suffering from an episode of major depression over a period of 12 months. Participants will be assessed at baseline and after 1, 3 and 6 months of follow up. At each time point, the following validated rating scales will be administered: Montgomery–Åsberg Depression Rating Scale (MADRS), Antidepressant Side-Effect Checklist (ASEC), EuroQual 5 Dimensions (EQ-5D), Short Blessed Test (SBT), and Charlson Age-Comorbidity Index (CACI). Outcome assessors and the statistician will be masked to treatment allocation. A total of 358 participants (179 in each group) will be enrolled.

**Ethics and dissemination:**

This study will fully adhere to the ICH E6 Guideline for Good Clinical Practice. Participants’ data will be managed and safeguarded according to the European Data Protection Regulation 2016/679. An external Ethical Advisory Board will help guarantee high ethical standards.

**Trial registration:**

Clinicaltrials.gov: NCT03779789, Registered on 19 December 2018. Submitted on 19 December. EudraCT number: 2018–001444-66.

**Trial status:**

Protocol version 1.5; 09/06/2018. Recruitment started In February 2019 and it is ongoing. It is expected to end approximately on 30 September 2021.

## Introduction

### Background and rationale

Depression is among the most disabling conditions worldwide [1]. It occurs in about 4% of older people in the community [2] and in up to 49% of persons admitted to nursing homes and hospitals [3, 4]. In older people, depression is associated with poor quality of life, reduced life expectancy, high risk of suicide [5], cognitive decline and dementia [6], reduced adherence to medical treatments and, therefore, poorer medical outcomes [7].

Current guidelines recommend pharmacological treatment in the case of moderate-to-severe depression [8]. Although different medications can be used in people with depression, the first-line treatment is typically antidepressants. Traditionally, the theory of a “chemical imbalance” (and particularly reduction in serotonin levels in some areas of the central nervous system) was claimed to explain the pharmacological mechanism of these drugs. However, there is growing evidence on multiple therapeutic targets of antidepressants, including neurotrophic actions and modulation of genetic, immunity and inflammatory processes. Selective serotonin reuptake inhibitors (SSRIs) are a class of antidepressants with the simplest pharmacological profile, and are generally chosen as a first-line treatment and are considered to represent a good balance between efficacy and safety in the general population, according to data from randomized trials [9]. However, in older people there is debate around the benefits and harms of antidepressants. Older people may be particularly vulnerable to adverse events due to frailties associated with the ageing process [10], medical comorbidities, multiple treatments and high risk of pharmacological interactions [11, 12]. SSRIs are generally considered the safest option in older people [13] and are therefore recommended by most guidelines as first-choice treatment [8, 14]. However, SSRIs are not without risks in older people; in particular, hyponatraemia, postural hypotension, falls, gastrointestinal bleeding and sexual dysfunctions are the most common side effects [15, 16]. Other antidepressants, such as tricyclic antidepressants (TCAs), serotonin and norepinephrine reuptake inhibitors (SNRIs) and mirtazapine are associated with a similar or higher risk of a number of adverse events, including sedation, confusion, urinary retention and cardiovascular and gastrointestinal issues [16], and are generally avoided in these patients. Moreover, the magnitude of beneficial effects of antidepressants in late-life depression has been questioned [17], making it hard for clinicians to accurately balance benefits and risks.

Vortioxetine is a novel antidepressant, licensed for the treatment of depression in 2013 by the US Food and Drug Administration (FDA) and the European Medicines Agency (EMA) [18, 19]. Vortioxetine is an antagonist to 5-HT3, 5-HT1D and 5-HT7 receptors, a partial agonist to the 5-HT1B receptor and a 5-HT1A receptor agonist [20, 21]. Its mechanism of action is not yet fully understood, but it is likely to be related both to direct modulation of the serotoninergic receptor activity and to inhibition of the serotonin transporter. Despite similarities with SSRIs, its pharmacological profile is claimed to be novel, particularly because of its stronger binding to the 5-HT1B receptor as compared to other SSRIs [22], and because of promising results in animal models, showing antipsychotic, antidepressant, and pro-cognitive activities (enhanced memory, cognition and executive functions) [23]. It is classified among “other antidepressants” by the World Health Organization (WHO) ATC/DDD Index 2018 [24]. Vortioxetine has similar pharmacokinetic properties in young and older adults [18], and current data suggest it should not adversely affect psychomotor or cognitive performance, wakefulness, body weight or electrocardiogram parameters [25, 26]. Further, possible beneficial effects on cognition emerged from three randomized trials in participants with cognitive impairment [27]. A recent Cochrane systematic review, which included 15 randomized trials (7746 participants), showed vortioxetine to be effective compared to placebo, while no significant differences emerged between vortioxetine and SNRIs as a class, in terms of both efficacy and tolerability [28]. The review did not include any study comparing vortioxetine with the SSRIs, but a recent network meta-analysis showed that vortioxetine is well-tolerated and effective when indirectly compared to SSRIs [9, 29]. Moreover, in two recent randomized trials, vortioxetine did not show significantly different effects on mood or cognitive performance when compared to paroxetine [30] and escitalopram [31], respectively. The only available trial conducted in the older people demonstrated that vortioxetine was more effective than placebo in terms of response, i.e. participants with > = 50% reduction in the Hamilton Depression Scale-24 total score in 8 weeks (301 participants, relative risk 1.49, 95% CI 1.14 to 1.95), while no differences emerged in terms of tolerability [32].

### Objectives

The study will assess if, under real-world clinical circumstances, vortioxetine is better-tolerated as compared with the SSRIs considered as a group in elderly participants with depression. In addition to tolerability, as secondary outcomes the study will assess acceptability, overall mortality, self-harm and suicide, adverse events, improvement of depressive symptoms, quality of life and cognitive performance.

## Methods and analysis

**FIg. 1:**
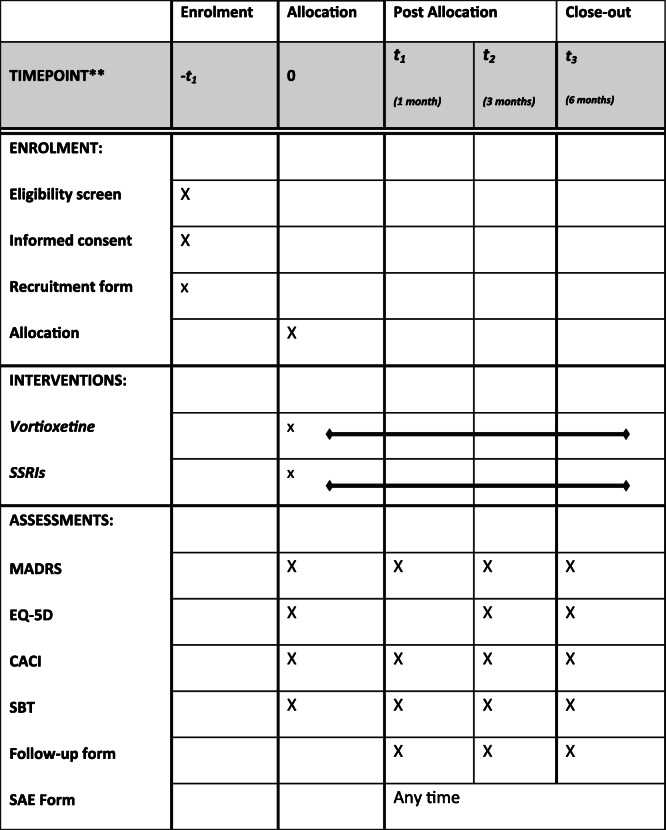
SPIRIT figure

This protocol has been reported according to the Standard protocol items: recommendation for interventional trials (SPIRIT) statement requirements. The complete SPIRIT checklist is available in Additional file 1 and in the table at the end of the manuscript (Fig. 1).

### Trial design

The Vortioxetine in the elderly vs SSRIs: a pragmatic assessment (VESPA) study is a randomized, parallel-group, multicentre, open-label, pragmatic, superiority trial. Over a 12-month recruitment period, psychiatrists from 13 Italian Psychiatric Services will consecutively enrol inpatients and outpatients aged 65 or years or older suffering from an episode of major depression and requiring treatment with an antidepressant. A threshold of 65 years of age was selected because this is generally accepted as the point of transition into older age [33] and has broadly been accepted as standard in previous randomized trials [29]. The study was designed in accordance with the principles of pragmatism, which aim to assess interventions under real-life circumstances by recruiting participants in ordinary care settings and employing outcomes with high practical value for clinicians [34].

**Fig. 2:**
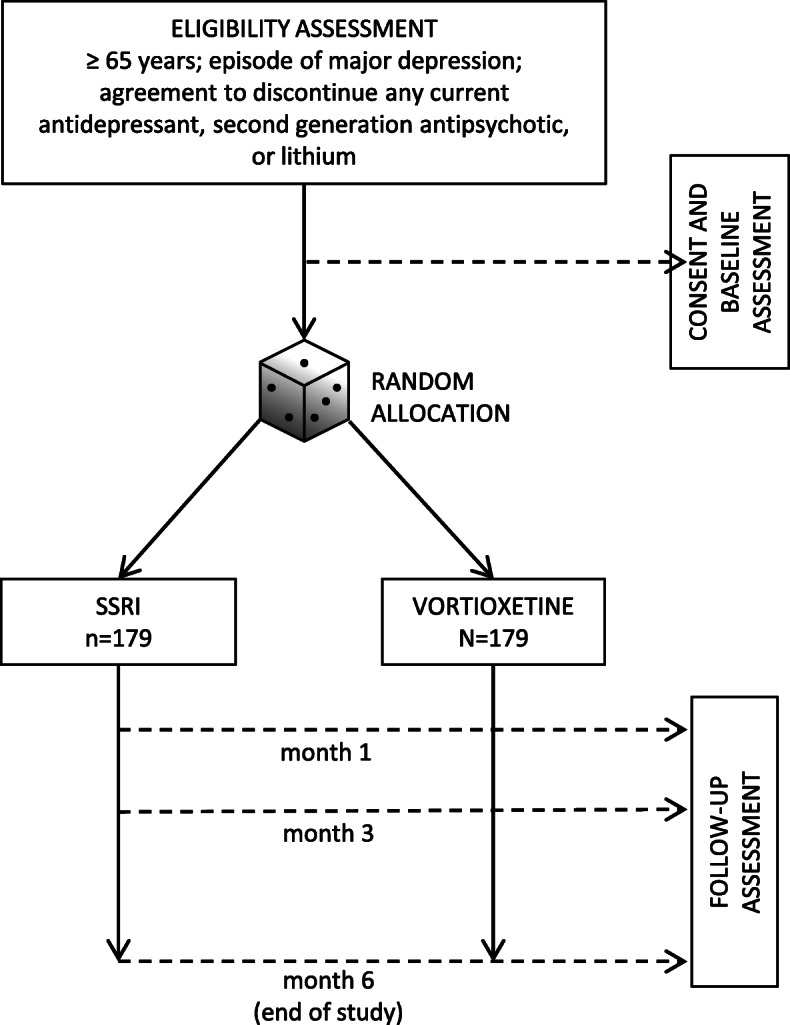
Study flow-chart. SSRI, selective serotonin reuptake inhibitor

Participants will be randomly allocated to vortioxetine or to one of the SSRIs. Apart from treatment allocation, clinicians and participants will be free to increase or decrease the dose according to clinical status and circumstances, and to stop or continue treatment as clinically indicated. Similarly, the use of concomitant medications during the study will be allowed according to clinical status and circumstances. Routine care outside the trial will continue as usual. Participants will be seen as often as clinically indicated during the study, with no extra visits required for the trial. The only requirement will be follow-up visits at 1, 3 and 6 months of follow up (Fig. 2). Visits will be conducted at the usual care facilities in each centre.

As a consequence of these pragmatic characteristics oriented to resemble clinical practice as much as possible, both participants and clinicians will not be blind to pharmacological treatments provided during the trial. Blinding will be applied to outcome assessors and statisticians performing the analyses. The study has been designed according to the principles described in the Consolidated standards of reporting trials (CONSORT) statement (extended version for pragmatic trials) [35] and in agreement with the SPIRIT 2013 statement [36] (see Additional file 1). The study is financially supported by the Italian Medicines Agency (AIFA - *Agenzia Italiana del Farmaco*) and has been approved by the Ethics Committee for Clinical Research of Verona and Rovigo (*Comitato Etico per la Sperimentazione Clinica delle Province di Verona e Rovigo*) (prot. n. 61,211 of the 19/09/2018; Protocol version n. 1.5 of the 09/06/2018).

### Assessment of pragmatism

**Fig. 3:**
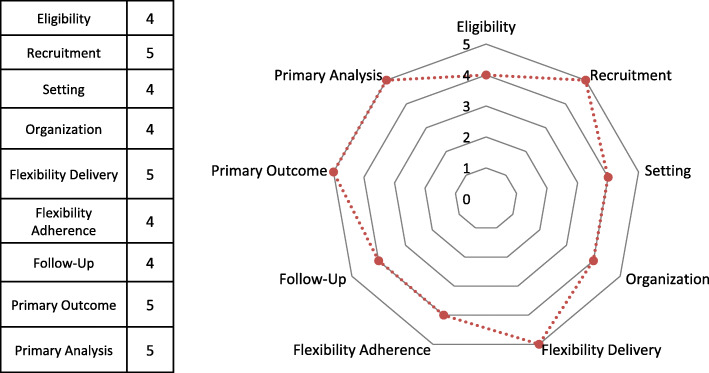
Pragmatism wheel according to the Pragmatic–explanatory continuum indicator summary-2 (PRECIS-2) tool

To quantify the level of pragmatism of our study, we employed the Pragmatic–Explanatory Continuum Indicator Summary-2 (PRECIS-2) [37]. This is a validated tool, developed to help investigators make design decisions consistent with the intended purpose of their trial. It explores nine domains (eligibility criteria, recruitment, setting, organisation, flexibility (delivery), flexibility (adherence), follow up, primary outcome and primary analysis), for each of which a score from 1 (very explanatory) to 5 (very pragmatic) is provided. The result is graphically summarized in Fig. 3.

Reasons for the scoring are reported in Table 1. Routine use of the PRECIS-2 tool when submitting randomized controlled trial (RCT) protocols to funders, research ethics committees and peer-reviewed journals, has increasingly been recommended, considering that not all RCTs self-labelled as “pragmatic” or “naturalistic” are actually pragmatic. This process can also help us understand the extent to which trial results may be relevant to real-world practice [38].

**Table 1:** Scoring using the PRECIS-2 tool

Items	Score	Rationale
**Eligibility -** to what extent are the participants in the trial similar to those who would receive this intervention if it was part of usual care	4	Target population: elderly with depression. Inclusion criteria are wide. No exclusion criteria will be applied in terms of setting of recruitment, severity of depression, past use of psychotropic drugs, current use of benzodiazepines, number and severity of medical comorbidities and multiple pharmacotherapies. Diagnoses are based on clinical judgment (considering DSM-5 criteria as a reference), as it is in usual practice
**Recruitment** - how much extra effort is made to recruit participants over and above what that would be used in the usual care setting to engage with patients?	5	Participants will be recruited without extra efforts. They will be recruited during usual appointments and/or visits
**Setting -** how different is the setting of the trial and the usual care setting?	4	The study is multicentre, based in more than 10 psychiatric centres of the National Health System in Italy with a University centre
**Organisation -** how different are the resources, provider expertise and the organisation of care delivery in the intervention arm of the trial and those available in usual care?	4	We will use usual staff and resources, but some extra resources will be necessary to hire researchers for the study. Visits will be conducted at the usual care facilities in each centre
**Flexibility (delivery) -** how different is the flexibility in how the intervention is delivered and the flexibility likely in usual care?	5	The intervention is flexible, similar to usual care
**Flexibility (adherence) -** how different is the flexibility in how participants must adhere to the intervention and the flexibility likely in usual care?	4	No extra measures. Participants will be free to partake in the intervention or drop it, but drugs will be prescribed and given to the participants during visits. This is different from usual care (patients have a prescription and go to the pharmacy to buy drugs)
**Follow up -** how different is the intensity of measurement and follow up of participants in the trial and the likely follow up in usual care?	4	The primary outcome will be assessed after 1, 3 and 6 months, as it is usually in everyday practice. Six months represent a clinically sound time frame for assessing the overall tolerability of medications, including both acute, short-term and medium-to-long-term effects. Visits could be slightly longer than usual to assess all the scales and long-term effects and adverse events could occur after 6 months
**Primary outcome -** to what extent is the trial’s primary outcome relevant to participants?	5	Primary outcome is relevant to participants and policy makers
**Primary analysis -** to what extent are all data included in the analysis of the primary outcome?	5	The intention-to-treat (ITT) population will consist of all randomized participants. This ITT population will be used for the analysis of both primary and secondary outcomes. Missing values in rating scales will be imputed using the last observation carried forward (LOCF) approach

The Pragmatic–Explanatory Continuum Indicator Summary-2 (PRECIS 5)-point Likert scale score: (1) very explanatory; (2) rather explanatory; (3) equally pragmatic/explanatory; (4) rather pragmatic; (5) very pragmatic

*DSM-5* the *Diagnostic and statistical manual of mental disorders, 5*^*th*^*edition*

### Eligibility criteria and study setting

The following inclusion criteria will be applied:

- The participant is 65 years of age or older;
- The participant is willing to participate by signing an informed consent form;
- The participant is suffering from an episode of major depression, according to the clinical judgment of recruiting psychiatrists, considering the *Diagnostic and statistical manual of mental disorders, 5*^*th*^*edition* (DSM-5) criteria as a reference standard;
- Treatment with an antidepressant is appropriate, based on clinical judgment;
- In case the participant is receiving pharmacological treatment, there is clinical agreement between investigator and participant to discontinue any of the following concomitant drugs: antidepressant, second generation antipsychotic or lithium. All other concomitant medications are allowed;
- Uncertainty about which trial treatment would be best for the participant.

Participants will be excluded in the case of:

- Dementia, of any type and stage, as formally diagnosed by a specialist (geriatrician, neurologist or other);
- Diagnosis of schizophrenia or bipolar disorder;
- Clinical conditions or treatments that contraindicate the use of oral vortioxetine or SSRIs, according to clinical/medical judgement (for example conditions or treatments that increase risk of bleeding, seizures, serotoninergic syndrome, hyponatraemia, etc.).

All medications will be prescribed according to routine clinical practice and current guidelines [8, 14], in compliance with the summary of product characteristics (SPC) registered in the AIFA databank (https://farmaci.agenziafarmaco.gov.it/bancadatifarmaci/home). Patients with a current depressive episode already receiving an antidepressant treatment that proved to be ineffective and/or poorly tolerated, and could benefit from the switch to vortioxetine or an SSRI, will be eligible for the study.

No exclusion criteria will be applied in terms of setting of recruitment, severity of depression, past use of psychotropic drugs, current use of benzodiazepines (as long as SPC indications are respected), number and severity of medical comorbidities and multiple pharmacotherapies. Such criteria will select participants similar to those who require antidepressant treatment under usual care, including patients with multiple medical comorbidities. The recruitment will be pragmatic, as participants will be consecutively enrolled among those attending inpatient and outpatient community services and fulfilling the study inclusion criteria. People with new-onset or pre-existing major depression will be eligible. Therefore, people who are not on antidepressant treatment at baseline and those who are already in treatment but needing a switch of medication on clinical grounds, will be eligible. There will not be any overt recruitment effort, as that could lead to the recruitment of participants who do not resemble usual care. Also, allowing different recruitment settings, having multiple sites of recruitment and selecting patients similar to those who are treated in every day clinical practice, will increase the generalizability of trial results. Randomization will be stratified by centre to control for potential risk of excessive heterogeneity between centres. All recruiters will take part in training meetings on recruitment procedures, including timing, how to administer rating scales and how to provide short synthetic yet exhaustive information to the patients. This is intended to minimize heterogeneous recruiting practices in different centres, which might reflect different characteristics of the patients recruited. We will keep track of the number of patients invited who refuse to participate. According to these features, the PRECIS-2 “setting” domain has been evaluated as pragmatic.

### Interventions

Participants will be randomized to either vortioxetine or one of the SSRIs. Doctors will be free to choose which SSRI is more appropriate among those marketed in Italy and commonly used in clinical practice in older people (sertraline, citalopram, escitalopram, paroxetine, fluoxetine, fluvoxamine). A flexible dosing schedule, within the licensed dose range and in line with the summary of product characteristics (SPC), will be suggested (Table 2) in order to resemble clinical practice as much as possible. All medications will be prescribed and switched accordingly to current guidelines.

**Table 2:** Treatments and dosing schedule

Medication	Licensed dose range in the elderly	Notes from the registered summary of product characteristics
Vortioxetine	5–20 mg/day	The minimum effective dose of 5 mg vortioxetine once daily should always be used as an initial dose for participants aged ≥ 65 years. Caution should be exerted when prescribing to elderly participants at doses > 10 mg vortioxetine once daily
Sertraline	50–200 mg/day	Caution is required in the elderly, because these patients may be at greater risk of hyponatraemia
Paroxetine	20–40 mg/day	In the elderly, increased plasma concentrations of paroxetine have been reported, however, within the range observed in younger patients. The treatment should start at the same doses used in adults
Citalopram	10–20 mg/day	In the elderly, half of the dose range prescribed in adults is required
Escitalopram	5–10 mg/day	In the elderly, half of the dose range prescribed in adults is required
Fluoxetine	20–60 mg/day	Caution is required when the dose is increased in the elderly, and generally the daily dose should not be above 40 mg/day. The maximum recommended dose is 60 mg/day
Fluvoxamine	100–300 mg/day	In elderly participants, titration should be slower and the dosage should always be established with caution

Clinicians and participants will choose the drug formulation (tablets versus drops) following everyday practice, and no measures will be implemented to optimise treatment adherence. According to the PRECIS-2 “flexibility-delivery” and “flexibility-adherence” domains, treatment delivery has been rated as pragmatic, although a full score of 5 could not be reached as we were formally required to follow the EU pharmacovigilance regulation [39, 40].

### Outcomes

The number of participants withdrawing from allocated treatment due to adverse events at the end of the study (6 months) will represent the primary outcome. This measure may be considered a pragmatic proxy of tolerability [41] as it occurs when adverse events actually reach an unbearable burden, as perceived by patients and/or relatives and/or carers and/or clinicians. Antidepressant treatment will be considered withdrawn due to adverse effects when the drug is stopped for more than two consecutive weeks following the occurrence of any adverse event, based on clinical judgement and/or as reported by participants. Participants will also be evaluated after 1, 3 and 6 months from randomization, collecting relevant clinical information and assessing scales, as shown in Table 3. The information on the primary outcome and on any co-medication taken by the participant will be collected using a predefined follow-up form. Side effects responsible for treatment withdrawal, and their severity, will be recorded on the follow-up form and an ad h*oc* form for severe adverse events (SAEs).

**Table 3:** Study timeline and tools

Procedures and tools	T0 Enrolment phase (duration: 12 months)	T1 (1 month)	T2 (3 months)	T3 (6 months)
Review of criteria for inclusion in the study	X			
Informed consent document signed	X			
Randomization (allocation to treatment and number assigned)	X			
Recruitment form	X			
ASEC		X	X	X
MADRS	X	X	X	X
EQ-5D	X	X	X	X
CACI	X	X	X	X
SBT	X	X	X	X
Follow-up form		X	X	X
Severe adverse event (SAE) form		←← any time →→		

*ASEC* Antidepressant Side-Effect Checklist, *MADRS* Montgomery–Åsberg Depression Rating Scale, *EQ-5D* Euroqol 5 Dimensions, *CACI* Charlson Age-Comorbidity Index, *SBT* Short Blessed Test

Secondary outcomes were chosen taking into account particular clinical characteristics of depression in older people (i.e. higher risk of committing suicide and higher likelihood of comorbid medical conditions) [42, 43] and the specific peculiar characteristics of vortioxetine (i.e. potential pro-cognitive effects) [23].

Secondary outcomes will include:

1. Acceptability: withdrawals from allocated treatment due to any cause (this outcome measure will include withdrawals due to side effects plus withdrawals for any other issues). Acceptability is generally considered a pragmatic measure of the balance between efficacy and tolerability of treatments [44];
2. Overall mortality;
3. Any episode of deliberate self-harm;
4. Suicide mortality;
5. Adverse events, measured as the mean change in scores on the Antidepressant Side-Effect Checklist (ASEC) [45] at each time point. ASEC is a validated rating scale measuring the occurrence and severity of 21 antidepressant adverse events;
6. Response to treatment, defined as a reduction of at least 50% in the baseline score of the Montgomery–Åsberg Depression Rating Scale (MADRS) [46, 47] at each time point. MADRS is a validated, 10-item questionnaire for assessing the severity of depression. Usually, scores of 0–6 indicate absence of depression, 7–9 mild depression, 20–34 moderate depression and > 34 severe depression. This rating scale is a reliable tool for assessing depression in elderly patients [48];
7. Efficacy, measured as mean change scores on the MADRS at each time point;
8. Quality of life, measured as mean change scores on the self-administered scale the Euroqol 5 Dimensions (EQ-5D) [49], at each time point. The EQ-5D explores 5 areas, including mobility, self-care, usual activities, pain/discomfort and anxiety/depression, and assesses the overall subjective perception of health on an analog scale. The EQ-5D is a reliable tool for assessing quality of life in older people [50];
9. Cognitive performance, measured as mean change scores on the Short Blessed Test (SBT) [51] at each time point. The SBT is a validated, six-item weighted instrument, originally designed to identify dementia, which assesses orientation, registration and attention.

Rating scales to assess the secondary outcomes will be administered by assessors blinded to treatment allocation, at 1, 3 and 6 months after randomization. In addition, the Charlson Age-Comorbidity Index (CACI) [52] will be employed. This is a validated rating scale used to evaluate the degree of medical comorbidity and predict 10-year survival in participants with multiple comorbidities. All study tools and phases are shown in Table 3.

### Safety

The VESPA study will operatively employ the definitions endorsed by the EC Directive 2001/20/EC [53]. As soon as a severe adverse event occurs, an ad hoc form for SAEs will be completed and forwarded to the coordinating centre (University of Verona), in accordance with the EU regulation about pharmacovigilance in clinical research [39]. If for any reason the disadvantages of participation appear to be significantly greater than foreseen, the Principal Investigator (CB) of the site will inform trial participants and the bodies providing ethical oversight.

Considering that the study medications are already in the Italian market, and considering that they will be prescribed for licensed indications without altering clinical practice, the VESPA study has not appointed an ad hoc data safety and monitoring committee.

### Randomization and assignment of interventions

Participants will be randomly assigned to vortioxetine or SSRIs in an allocation ratio of 1:1. A centralized web-based randomization procedure will be employed to guarantee the concealment of allocation. The trial biostatistician will prepare the sequence of treatments randomly permuted in blocks of constant size. The site investigators will not know the block size. Allocation will be stratified by recruiting centre. By using the web-based application RedCap [54], investigators will be able to screen participants for inclusion, administer instruments maintaining blinding to treatment allocation and randomize the participants.

### Data collection and management

At baseline, before randomization, and after 1, 3 and 6 months, socio-demographic and clinical information will be collected along with the administration of the aforementioned validated rating scales (the MADRS, EQ-5D, CACI, SBT and ASEC). Data on the use of other medications will be registered at every visit. The ASEC scale will be administered only during follow up.

All study data will be collected with RedCap and digitally stored by the *Istituto di Ricerche Farmacologiche Mario Negri IRCCS*, a not-for-profit biomedical research organization based in Milan (Italy), where the statistical analysis will also be performed. RedCap will allow immediate data validation at the time of data collection. Moreover, a set of electronic and manual edit checks will be performed. The local coordinator of each recruiting centre will store and safely preserve hard-copy documents (signed informed consent and self-administered questionnaires) for at least 7 years after the end of the study, according to the Italian law. At the end of the study the full dataset will be made available upon motivated request, as a spreadsheet file in an online repository (e.g. Dryad Digital Repository). This is in line with findable, accessible, interoperable and reusable (FAIR) principles [55], aimed at enhancing the accessibility and reutilization of novel research data.

The accuracy and completeness of data collection will be monitored by site visits. At least one visit for each recruiting centre is planned. Furthermore, auditing will also be carried out remotely, as the data manager of the study will be able to regularly check the trial dataset through the web application RedCap.

### Sample size

Based on differences in the withdrawal rate due to adverse events among patients receiving SSRIs or vortioxetine, as identified in a meta-analysis of the use of antidepressants in older people [13] and in three clinical trials of vortioxetine in older patients with depression [26, 32, 56], we expect the vortioxetine group to have a clinically significant advantage, with a reduction in the withdrawal rate from about 17% [13] to about 5% [26, 32, 56]. A sample size of 276 participants (138 in each group) achieves 90% power to detect a difference of 12% between the two proportions of patients who withdraw, in favour of vortioxetine. The test statistics will be the two-sided *Z* test with pooled variance. The significance level of the test is targeted at 5%. On the basis of the aforementioned studies, we can assume that about 23% of the participants could withdraw within 6 months (the mean of the total dropout rates in vortioxetine and SSRI studies in older people). Therefore 358 participants (179 in each group) will be enrolled in order to obtain at least 276 evaluable participants. The sample size calculation was performed according to the methodology described by Pocock [57].

### Statistical analysis

According to the pragmatic principle of intention to treat (ITT), efforts will be made to follow each participant until the end of the study. The ITT population will consist of all randomized participants, and will be used for the analysis of both primary and secondary outcomes. The absolute risk of the primary outcome will be calculated in the ITT population. Participants with missing primary outcome data will be allocated to the worst outcome. When possible, in addition to the primary analysis, appropriate statistical methods will adjust for the potential confounding effect of prognostic factors (sex, age, living condition, severity of comorbid medical conditions, previous psychiatric history, MADRS score at baseline). Missing rating scale scores will be imputed using the last observation carried forward (LOCF) approach: ratings will be carried forward from the last available assessment to the 6-month follow-up assessment. As a secondary analysis, missing scores will be imputed following a multiple imputation approach [58].

In order to check the results of the ITT approach, though for confirmatory purposes only, the primary outcome will also be analysed using a per-protocol (PP) approach. According to the PP approach, analysis will be restricted to participants with primary outcome assessment available at 6 months. Participants withdrawing for reasons not related to adverse effects will be excluded from the analysis.

The proportion of participants withdrawing from the study due to adverse events within 6 months of follow up will be compared between the two groups of treatment using a logistic regression with centre (random variable) as a covariate. A multivariable analysis (secondary analysis) will be performed through a Poisson regression model with robust error variance, given that this procedure allows direct estimation of relative risk [59].

For dichotomous secondary outcomes, the proportion of participants who withdraw from the study within 6 months due to adverse events will be compared between the two groups of treatment using logistic regression with centre (random variable) as a covariate. When possible, a multivariable analysis will be performed through a Poisson regression model with robust error variance. For continuous secondary outcomes, the 6-month estimate will be compared between the two treatment groups using analysis of covariance with baseline value as an additional covariate, or using the Mann-Whitney test on the data on changes, according to the distribution of the variable. The same outcomes will be studied using linear mixed models, taking into account all assessments to evaluate the rate of change with shorter repeated evaluations and no need for imputation of missing values. Further, score changes on the subscales will be evaluated in order to detect possible specific treatment-related side effects.

A Cox proportional hazard model will be used to explore time to treatment withdrawal due to adverse events (secondary analysis). The proportional hazard assumption of the effects will be tested. A Cox proportional hazards model was chosen as it is a regression model commonly used for investigating the association between the survival time of patients (who drop out due to side effects) and one or more predictor variables (allocation to vortioxetine or control SSRI).

Adverse events will be tabulated. The nominal value for statistical significance will be set at 0.05, two-tailed. A specific statistical analysis protocol will be produced and made publicly available before the inclusion of the last participant. All analyses will be performed using STATA [60], release 15 or higher.

## Ethics and dissemination

This study will be conducted according to globally accepted standards of good clinical practice, as defined in the ICH E6 Guideline for Good Clinical Practice, 1 May 1996, in agreement with the Declaration of Helsinki [61] and in keeping with local regulations. The recruiting investigators will obtain informed consent. All participants will be informed about the study procedures and aims, both verbally and by written documentation. The subject’s consent will be confirmed by the personally dated signature of the participant and by the personally dated signature of the person conducting the informed consent discussion. Participants can withdraw from the study at any time without further explanation or any negative consequences. Participants’ data will be managed and safeguarded in accordance with the European Data Protection Regulation 2016/679 [62]. The highly pragmatic design will minimize the time deduction to ordinary clinical practice. An Ethics Advisory Board (EAB) will indirectly supervise the process of recruitment, informed consent procedures and data management (protection and privacy), taking into due account the vulnerability of the population. Once the final report is available, the study results will be extensively disseminated to the international scientific community in the form of peer-reviewed journal articles, giving preference to open-access journals.

The study is financially supported by the AIFA and has been approved by the Ethics Committee for Clinical Research of Verona and Rovigo (*Comitato Etico per la Sperimentazione Clinica delle Province di Verona e Rovigo*) (prot. n. 61,211 of the 19/09/2018; Protocol version n. 1.5 of the 09/06/2018).

## Discussion

This study is designed to achieve a high level of pragmatism. This approach will allow us to minimize the risk of selection bias (particularly relevant when assessing frail populations such as older people, who are often excluded from experimental research), to resemble routine clinical procedures as much as possible and therefore to maximize the external validity and generalizability of the results [34]. First, participants will be enrolled on the basis of the need for an antidepressant prescription because of a depressive episode. Although the diagnostic reference standard is represented by DSM-5 criteria, in line with routine clinical practice, no formal diagnostic assessment will be performed, in order to recruit patients as similar as possible to those treated in the real world. No limitations to the recruitment setting will be applied. Rating scales will be easy to administer and of relatively short duration, in order not to substantially alter clinical practice. Second, a web-based application will allow us to simplify the process of recruitment, randomization and collection of socio-demographic and clinical data, minimizing the time deducted from ordinary clinical practice. Third, the comparison group will consist of participants receiving any of the available SSRIs. We made this choice in order to avoid the possibility of selection bias, that is to avoid the systematic exclusion of participants who did not benefit from a specific SSRI in the past. Furthermore, a flexible dosing schedule will be employed, according to clinical judgement, within the recommended therapeutic range.

Some limitations need to be outlined. First, according to the current pharmacovigilance regulation of the European Union, medication boxes must be labelled and dispensed by the hospital pharmacy. This deviates from ordinary practice and may have an impact on adherence to medications, since participants will not have to go and buy medications at their local pharmacies, but the medications needed until the next visit will be distributed to them after each visit. Second, in order to avoid the potential confounding effect of other psychotropic drugs, to be included in the trial patients have to discontinue any other antidepressant or second generation antipsychotic before random allocation, but after random allocation any concomitant medication will be allowed. Again, the aim of this is to reflect everyday practice, as older patients are sometimes prescribed low doses of second-generation antipsychotics or antidepressants (e.g. mirtazapine, amitriptyline, trazodone) for treatment of insomnia or for other symptoms (e.g. cachexia, cephalalgia, etc.).

Third, the open-label design might be associated with a risk of performance bias. Theoretically, it may be possible that clinicians, being aware of the treatments received by participants, perform differently in the allocated treatment arms, based on personal subjective judgements. For example, they may provide vortioxetine or the control SSRI at excessively low or high doses, altering the likelihood of participants dropping out of treatment because of side effects or lack of efficacy. Although we cannot completely rule out this possibility, we note the following. First, as both treatment arms involve active antidepressants, it seems unlikely that doctors involved in the study, working in very diverse settings across Italy, share similar a priori opinions and, based on these opinions, systematically favour or disfavour either vortioxetine or the control SSRIs. Second, information on any dose changes, use of concomitant medicines and provision of additional non-pharmacological treatments will be recorded, which is important to investigate if the two groups were treated similarly, apart from the study medications. Third, blinded assessors will independently assess the presence and severity of adverse events using the ASEC, and this will allow an internal quality check of the accuracy of the primary outcome.

Considering the overall psychological, medical and economic burden of depression in older people, and the few available pharmacological alternatives for treating them, the results of this study are likely to have a positive impact on everyday clinical practice. Furthermore, considering the pragmatic nature of the study, we expect that results will be immediately applicable to ordinary practice without requiring any specific training or implementation strategies. If the hypothesis of better tolerability of vortioxetine is confirmed, this drug may become a reference first-line drug for the treatment of depression in older people. This, besides improving the overall psychological well-being and quality of life in older people with depression, might at the same time reduce hospitalizations for medical adverse events (such as falls, bleeding, hyponatraemia, QTc alterations), poor medical outcomes and related healthcare costs. If, on the other hand, vortioxetine is not better-tolerated than SSRIs, its place in the treatment of older people will be clearer, and the VESPA study results will be used to better inform clinical and policy practice.

Additionally, this study may have regulatory implications, considering that currently according to the EMA, “caution is advised when treating participants ≥ 65 years of age with doses higher than 10 mg vortioxetine once daily for which data are limited” [19, 21]. We expect that this statement may be reformulated in view of the study results: if vortioxetine is better-tolerated than the SSRIs, by mentioning its favourable tolerability profile; if vortioxetine is less well-tolerated than the SSRIs, by further reinforcing the cautionary statement.

## Supplementary information

**Additional file 1.** SPIRIT 2013 Checklist: Recommended items to address in a clinical trial protocol and related documents.

## Data Availability

At the end of the study the full dataset will be made available upon motivated request, as a spreadsheet file in an online repository (e.g. Dryad Digital Repository). This is in line with FAIR principles [55], aimed at enhancing the accessibility and reutilization of novel research data.

## Abbreviations

AIFA - *Agenzia Italiana del Farmaco*: Italian Medicines Agency
ASEC: Antidepressant Side-Effect Checklist
CACI: Charlson Age-Comorbidity Index
EAB: Ethics Advisory Board
EMA: European Medicine Agency
Euroqol-5 D: Euroqol 5 Dimensions
FDA: Food and Drug Administration
FAIR: Findable, accessible, interoperable and reusable
ITT: Intention to treat
LOCF: Last observation carried forward
MADRS: Montgomery–Åsberg Depression Rating Scale
PP: Per-protocol
PRECIS-2: Pragmatic–explanatory continuum indicator summary-2
RCT: Randomized controlled trial
SAE: Severe adverse events
SBT: Short Blessed Test
SNRIs: Serotonin and norepinephrine reuptake inhibitors
SPC: Summary of product characteristics
SSRIs: Selective serotonin reuptake inhibitor
TCAs: Tricyclic antidepressants
VESPA: Vortioxetine in the elderly vs SSRIs: a pragmatic assessment
WHO: World Health Organization
